# Molecular Mechanisms and Clinical Applications of Neural Regeneration Through Dental Pulp Stem Cells

**DOI:** 10.1155/sci/8069882

**Published:** 2026-04-08

**Authors:** Xinxuan Wang, Baicheng Yi

**Affiliations:** ^1^ Stomatology Centre, Southern Medical University Shenzhen Hospital, Shenzhen, Guangdong, China, fimmu.com; ^2^ School of Stomatology, Shenzhen University, Shenzhen, Guangdong, China, szu.edu.cn; ^3^ Department of Stomatology, Shenzhen University General Hospital, Shenzhen, Guangdong, China, szu.edu.cn

**Keywords:** anti-apoptosis, biomaterial scaffolds, dental pulp stem cells, nerve regeneration, neuronal differentiation, neurotrophic factors, paracrine effects, regenerative medicine

## Abstract

Neural injuries affecting both the central nervous system (CNS) and peripheral nervous system (PNS) pose a great clinical challenge due to the neural tissue’s limited self‐regenerative capacity. Human dental pulp stem cells (hDPSCs), derived from the neural crest and easily obtained from extracted teeth, exhibit considerable potential for neural regeneration. This potential is attributed to their ability to directly differentiate into various neuronal cell types, paracrine effects, and interactions with biomaterial scaffolds. In this review, we reviewed the molecular mechanisms by which hDPSCs support neural repair, highlighting their direct neuronal differentiation function, neuroprotection function via paracrine signaling, and recent innovations in biomaterial scaffolds that enhance the viability of hDPSCs for neuroregenerative applications. Preclinical studies have shown promising therapeutic effects of hDPSCs in spinal cord injuries (SCI), strokes, Parkinson’s disease (PD), Alzheimer’s disease (AD), and peripheral nerve injuries. However, challenges remain, including optimizing neuronal differentiation specificity, ensuring immunological safety, and achieving scalable clinical applications. Future research should focus on standardizing manufacturing protocols, implementing strict quality control, and developing functional assays linked to neural recovery to maximize the potential of hDPSCs for nervous system regeneration.

## 1. Introduction

### 1.1. The Significance and Challenges of Neural Regeneration

Nervous system injuries, particularly those to the central nervous system (CNS) and the peripheral nervous system (PNS), still pose great challenges for clinical neuroscience in modern days [1–4]. Most CNS and PNS are a result of injuries due to trauma, tumors, infections, autoimmune diseases, vascular events, neurodegeneration, and metabolic disorders [5–8]. Due to the limited regenerative capacity of nervous tissue, CNS injuries are nearly irreparable, while peripheral nerve injuries recover slowly and often with limited functional recovery [3, 9, 10]. Neural injuries frequently result in permanent motor, sensory, cognitive, and multisystem dysfunction, systemic immunosuppression, and long‐term complications [11–13]. They dramatically affect patients’ work capacities, mental health and become significant determinants of quality of life in improving chronic disease settings [14, 15].

Clinical treatment options for neural injuries mainly include surgical suturing, nerve transplantation, pharmacological release of growth factors, and electrostimulation [16–21]. Although mild to moderate nerve damage, such as nerve compression or rupture, may achieve partial recovery with clinical treatments, therapeutic outcomes remain limited in cases of severe or extensive injury [22]. Traditional clinical interventions face numerous limitations, including limited availability of donor nerves, high invasiveness, poor target specificity, low tissue compatibility, slow regeneration rates, inadequate axonal guidance, transient effects of pharmacological interventions, and immune rejection [2, 23–27].

With advances in regenerative medicine and stem cell biology research, stem cells, with their self‐renewal and multilineage differentiation potential, offer new approaches for neural tissue repair [2]. Researchers have increasingly focused on human dental pulp stem cells (hDPSCs), which are noninvasive, easy to collect, and low in immunogenicity [28, 29]. Recent research has increasingly focused on the neural lineage differentiation of hDPSCs and their application in treating neurological disorders, both in vivo and in vitro. This is particularly evident in studies utilizing advanced delivery systems and novel biomaterials to enhance the efficiency and precision of neural regeneration by hDPSCs [30].

### 1.2. Advances and Breakthroughs of Stem Cell Therapy in Neural Regeneration

With the rapid advancement of regenerative medicine, stem cell therapy has increasingly emerged as a promising strategy for treating neurological disorders [31]. Stem cells possess remarkable self‐renewal capacity, multilineage differentiation potential, immunomodulatory and neurotrophic effects. Stem cell therapy is considered one of the approaches capable of overcoming the limitations of traditional drug or surgical treatments. This potential has drawn significant attention in both basic research and clinical translation, making it an increasingly important direction driving research on neural tissue regeneration [32]. The primary stem cell types currently used in neural regeneration research include embryonic stem cells (ESCs), induced pluripotent stem cells (iPSCs), neural stem cells (NSCs), and mesenchymal stem cells (MSCs) [33–36]. Among these, ESCs and iPSCs possess high pluripotency and can differentiate into mature, fully functional neurons. However, their clinical application remains constrained by ethical controversies, potential tumorigenic risks, and immune rejection [37].

NSCs inherently possess neurogenetic differentiation potential, enabling efficient differentiation into neurons, astrocytes, and oligodendrocytes. These differentiated cells stably express mature neuronal markers such as microtubule‐associated protein 2 (MAP2) and tubulin beta 3 class III (TUBB3), while generating action potentials (APs). However, under pathological conditions, NSCs tend to differentiate into glial cells rather than neurons [38, 39]. NSCs’ neurodifferentiation is regarded as the “gold standard” in neural regeneration research. However, the acquisition of NSCs typically relies on sourcing fetal brain tissue or inducing them from iPSCs. These procedures involve ethical controversies or face operational limitations [40]. Allogeneic transplant recipients often require immunosuppressive therapy, and cells undergoing long‐term in vitro expansion may exhibit instability, such as abnormal proliferation or tumor formation. These factors collectively limit the clinical application prospects of NSCs [41].

In contrast, MSCs possess characteristics such as diverse sources, simple acquisition methods, and low immunogenicity, making them one of the best stem cell types for clinical translation. MSCs can be isolated from bone marrow, adipose tissue, umbilical cord, and dental pulp. It has been demonstrated to have good safety and therapeutic potential in multiple neurological disease models [42]. Among human MSCs, hDPSCs have garnered significant attention due to their unique neural crest origin. hDPSCs can be obtained non‐invasively from clinically discarded tissues (e.g., supernumerary teeth, impacted wisdom teeth, orthodontic extractions), offering abundant sources, straightforward procedures, minimal ethical concerns, and high accessibility. As a neural crest‐derived MSC lineage, hDPSCs exhibit a more pronounced tendency toward neural cell differentiation compared to bone marrow‐derived (BMSCs) and adipose‐derived MSCs (ADSCs). hDPSCs demonstrating higher expression levels of neural differentiation markers such as TUBB3 and MAP2 [43]. hDPSCs secrete higher levels of neurotrophic factors, including nerve growth factor (NGF), brain‐derived neurotrophic factor (BDNF), vascular endothelial growth factor (VEGF), neurotrophin‐3 (NT‐3), and glial cell‐derived neurotrophic factor (GDNF). These factors play a crucial role in promoting neuronal survival and axonal outgrowth [43–46]. hDPSCs possess potent immunomodulatory capabilities. Compared to BMSCs, hDPSCs suppress the proliferation of activated T cells more effectively and secrete higher levels of immunomodulatory and anti‐inflammatory factors, including TGF‐β1, HGF, IL‐10, and IL‐13 [47]. Additionally, hDPSCs secrete multiple factors promoting angiogenesis and neuroprotection, demonstrating outstanding efficacy in treating immune‐mediated diseases and neurological injuries [48]. hDPSCs exhibited a 91% inhibition of T cell proliferation compared to 75% for strong bone marrow MSCs, suggesting a higher immunosuppressive potential [49]. hDPSCs exhibit superior long‐term stability. Derived from the neural crest, hDPSCs maintain neural progenitor cell characteristics even in an undifferentiated state. Following neural induction, they sustain expression of neuro‐associated markers and neurotrophic functions for extended periods and persist in vivo for weeks. BMSCs can only acquire relatively stable neural or neural precursor phenotypes in the early stages, with insufficient evidence for long‐term stable differentiation and functional integration [44, 50, 51]. ADSCs typically exhibit transient neurite‐like phenotypes but readily dedifferentiate over time. Their expression of neurogenic markers and functional stability is often inadequate, with long‐term effects primarily attributed to neurotrophic factor secretion rather than genuine, stable neural differentiation [45, 52].

### 1.3. Characteristics of hDPSCs

hDPSCs represent a unique group of MSCs that are obtained from the dental pulp tissue [53]. They are characterized by adherent growth, possess the potential for multilineage differentiation, and express specific MSC surface markers such as CD73, CD90, and CD105, along with various additional markers including CD27, CD29, CD44, CD146, CD166, CD271, and STRO‐1 [27, 48, 54–56]. The expression of these markers is observed not only in hDPSCs but also in MSCs sourced from a variety of other tissues. In contrast, neither MSCs nor hDPSCs express surface markers such as CD45 (for hematopoietic cells), CD14 (for monocytes/macrophages), CD19 (associated with B cells), or major histocompatibility complex class II (MHC‐II) [54–57]. The biological properties of hDPSCs are demonstrated by their ability to differentiate into multiple cell types, including neurons, osteoblasts, adipocytes, chondrocytes, and endothelial cells [58]. In contrast to other sources of MSCs, dental pulp tissue is derived from the neural crest [59]. Neural crest cells undergo an epithelial‐mesenchymal transition, acquiring migratory capabilities that allow them to extensively travel to various regions of the embryo, including the pharyngeal arches, which serve as precursors for tissues and organs associated with craniofacial structures [60, 61]. Some neural crest stem cells can evolve into MSCs, contributing to the formation of mesenchyme that originates from neural crest cells, specifically ectomesenchyme. This mesenchyme subsequently gives rise to oral connective tissue, as well as cartilage, muscle, and bone. Even in the absence of neural induction, hDPSCs can express markers associated with both neural progenitor cells and mature neurons [62]. Key markers related to neural development include Nestin, TUBB3, p75 neurotrophin receptor, and neurofilament proteins [63, 64]. Compared to dental follicle and dental papilla, dental pulp‐derived stem cells exhibit greater neurogenic differentiation potential and represent a more suitable stem cell source for applications in neurodegenerative diseases [65]. These findings suggest that hDPSCs have significant potential for neural differentiation.

## 2. Mechanisms Underlying Neural Regeneration Using hDPSCs

**Figure 1:**
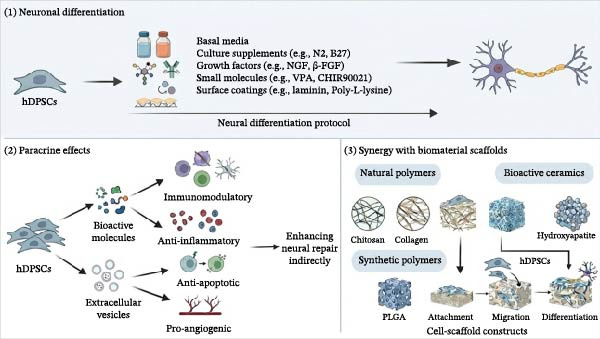
Mechanisms of neural regeneration mediated by hDPSCs. This schematic illustrates the three major mechanisms through which hDPSCs contribute to neural regeneration. (1) Neuronal Differentiation: hDPSCs undergo neural differentiation via protocols involving basal media, culture supplements (e.g., N2, B27), growth factors (e.g., NGF, basic fibroblast growth factor, β‐FGF), small molecules (e.g., VPA, CHIR99021), and surface coatings (e.g., laminin, poly‐L‐lysine). (2) Paracrine Effects: hDPSCs secrete bioactive molecules and extracellular vesicles that mediate immunomodulatory, anti‐inflammatory, antiapoptotic, and pro‐angiogenic effects, enhancing neural repair indirectly. (3) Synergy with Biomaterial Scaffolds: Natural and synthetic biomaterials provide structural support for hDPSC attachment, migration, and differentiation. These include polymers such as chitosan, collagen, PLGA, and bioactive ceramics. This schematic was generated using Nano Banana2 and manually verified for accuracy by the authors.

hDPSCs exhibit significant potential for neural regeneration through multiple synergistic mechanisms, including directed neuronal differentiation, paracrine signaling effects, and interaction with biomaterial scaffolds (Figure 1). To provide a systematic overview of the progress in hDPSCs neurogenic differentiation, Table 1 summarizes the landmark studies and pivotal findings regarding the mechanisms and advancement of this field.

**Table 1:** Landmark studies and pivotal findings regarding the mechanisms of hDPSCs’ neurogenic differentiation.

Authors	Key findings	Basal medium	Induction factors	Scaffold/culture	Efficiency	Electrophysiology
Arthur et al. [66]	Functional sodium currents were first detected in hDPSC‐derived cells via electrophysiological analysis	Neurobasal‐A DMEM/F‐12	β‐FGF, EGF, B27 ITS, retinoic acid	2D, coated with polyornithine and laminin	≥70% positive cells (TUBB3, PSA‐NCAM, NF‐M, NF‐H)	~8.5‐fold increased inward current; TTX‐sensitive functional voltage‐gated Na^+^ channels (Na^+^‐mediated)
Király et al. [67]	Developed a 3‐step protocol to differentiate hDPSCs into functional neurons with Na^+^/K^+^ channel activity and neural marker expression	Neurobasal‐A DMEM/F‐12	5‐azacytidine, β‐FGF, TPA, IBMX, Forskolin, dbcAMP, NT‐3, ITS, N2, B27, NGF, ITS	2D, coated with poly‐l‐lysine	40% of cells survived, displayed complex neuronal morphology (bipolar/stellate forms) and expressed neuronal markers	Exhibits sodium currents and potassium currents
Gervois et al. [68]	Depolarization events resembling action potentials	DMEM/F‐12	B27, β‐FGF, EGF	2D, coated with poly‐L‐ornithine (PLO) and Laminine	Significant increase	Evoked action potentials
Heng et al. [69]	Chemical induction enhance neurogenic differentiation	DMEM/F‐12, Neurobasal	Valproic acid, CHIR99021, Repsox, Forskolin, SP600125, GO6983, Y‐27632 and Dorsomorphin	2D	Increased MAP2, NeuN, NSE, NFM	Higher calcium transient (F/Fo) peaks
Chang et al. [70]	Directed differentiation into dopaminergic and motor neuron‐like cells	DMEM/F‐12	SHH, FGF8, retinoic acid (RA), N2	2D induction culture	Increased TH^+^ and subtype‐specific marker expression (partial quantification reported)	Not performed
Rafiee et al. [71]	EGF/β‐FGF enhanced neural progenitor and neuronal marker expression	DMEM/F‐12	EGF, β‐FGF, N2, IBMX, Indomethacin	Neurosphere‐like formation	Increased Nestin and MAP2 positive cells	Not performed
Feng et al. [72]	3D porous chitosan scaffolds enhance differentiation	DMEM/F‐12	N2, B27, β‐FGF, NGF, BDNF	Chitosan porous scaffolds	12% MAP2	Not performed
Al‐Maswary et al. [62]	ATRA and BDNF promoted neuronal maturation‐related gene expression	DMEM/F‐12	ATRA, BDNF	2D collagen‐I coated	Upregulation of TUBB3, NF‐M, GAP43, SYN1	Acquired measurable *I*_Na_ and *I*_Kss_

Abbreviations: 5‐aza, 5‐azacytidine; ATRA, all‐trans retinoic acid; B27, B‐27 supplement; β‐FGF, basic fibroblast growth factor; dbcAMP, dibutyryl cyclic adenosine monophosphate; DMEM/F‐12, Dulbecco’s modified Eagle medium/F‐12; EGF, epidermal growth factor; FGF8, fibroblast growth factor 8; Forskolin, forskolin; IBMX, 3‐isobutyl‐1‐methylxanthine; IBMX, 3‐isobutyl‐1‐methylxanthine; ITS, insulin–transferrin–selenium; MAP2, microtubule‐associated protein 2; N2, N‐2 supplement; NeuN, neuronal nuclei antigen; NF‐H, neurofilament heavy chain; NF‐M, neurofilament medium chain; NGF, nerve growth factor; Neurobasal‐A, Neurobasal‐A medium; NT‐3, neurotrophin‐3; PSA‐NCAM, polysialylated neural cell adhesion molecule; RA, retinoic acid; SHH, sonic hedgehog; TH, tyrosine hydroxylase; TPA, 12‐O‐tetradecanoylphorbol‐13‐acetate; TTX, tetrodotoxin; TUBB3, neuron‐specific class III beta‐tubulin; VPA, valproic acid.

### 2.1. Neuronal Differentiation

Various in vitro neural induction strategies for hDPSCs employ combinations of basal medium, culture supplements, growth factors, small molecules, and distinct culture coatings to modulate the differentiation process.

① Basal medium. The initial study on the neuro‐induction of stem cells derived from dental tissues was conducted by Arthur and colleagues. Their research demonstrated that neural induction of hDPSCs using Neurobasal‐A as the primary medium led to increased expression of neuron‐associated markers, including neural cell adhesion molecule (NCAM), neurofilament‐M (NF‐M), and neurofilament‐H (NF‐H) [66]. Several earlier studies utilized Dulbecco’s modified Eagle medium (DMEM) and Alpha minimum essential medium (αMEM) as the basal medium for neural induction in hDPSCs [73]. Recently, most studies utilized Neurobasal‐A, DMEM/F‐12, and neurobasal medium [74, 75]. The latest study showed that serum‐free media such as NeuroCult^TM^ can reduce the odontogenic differentiation tendency of hDPSCs and enhance neural differentiation efficiency. Moreover, this media has no animal‐derived components, which improves its clinical applicability [76].
② The addition of culture supplements during the neuronal differentiation of hDPSCs can improve differentiation efficiency, reduce the proportion of undifferentiated cells, and enhance the generation of functional neurons. N2 and B27 are commonly used supplements. The N2 supplement was originally developed by Bottenstein and Sato in 1979. It provides a chemically defined, serum‐free culture environment for neural progenitor cells, supporting cell proliferation, promoting axonal growth, and enhancing neural differentiation [77]. B27 is another classic supplement commonly used for serum‐free neuronal culture. It was developed by Brewer et al. in 1993 to optimize the survival and functional maintenance of hippocampal neurons under serum‐free conditions. B27 can inhibit the proliferation of glial cells, ensure neuronal nutrition, and enhance neuronal survival [78]. Insulin–transferrin–selenium (ITS) is a three‐component supplement developed by Bottenstein et al. in 1979 to enable serum‐free culture of neuroblastoma cells. It provides essential metabolic support to cells and synergistically promotes neuronal differentiation, neurite outgrowth, and functional connectivity [79, 80]. In earlier studies, fetal calf serum (FCS) was also chosen to be added to the neuronal induction medium for hDPSCs [67, 81]. In recent years, driven by clinical translation demands, researchers have increasingly focused on developing serum‐free culture media supplemented with defined components to achieve safe, controllable, and reproducible translational approaches. These culture supplements can synergise with serum‐free media to enhance the neural differentiation potential of hDPSCs [82].
③ Growth Factors. Neurotrophic factors, such as BDNF, NT‐3, and GDNF, as well as embryonic developmental signaling molecules such as NGF, bone morphogenetic protein 2 (BMP‐2), bone morphogenetic protein 4 (BMP‐4), and sonic hedgehog (SHH), also play important roles as “growth factors” in the development and regeneration of the nervous system [83–85]. NGF and β‐FGF have been shown to synergistically promote the differentiation of hDPSCs into neuron‐like cells, accompanied by upregulated expression of neural markers [86]. Sirt1 acts as a crucial upstream regulator that modulates the activation of ERK/MAPK and AKT signaling during NGF/(β‐FGF)‐induced neural differentiation [87]. NGF andβ‐FGF synergistically promote the differentiation of hDPSCs into neural‐like cells and enhance the expression of neural markers [86, 87]. This process is closely associated with the ERK and AKT signaling pathways, which regulate neural differentiation [86]. In addition, when used in combination with β‐FGF, epidermal growth factor (EGF) can also induce the differentiation of hDPSCs into neural progenitor cells, which are characterized by the expression of neural‐specific markers such as Sox1, Pax6, and NF‐M. This effect is particularly prominent in rapidly dividing subpopulations of hDPSCs [88].
④ Small molecules. Certain small‐molecule compounds with epigenetic effects and the ability to activate or inhibit key signaling pathways can also serve as components of neural induction protocols for hDPSCs. These small molecules include the histone deacetylase inhibitor valproic acid (VPA), the TGF‐β pathway inhibitors Repsox and SB431542, the GSK‐3β inhibitor CHIR99021, the cAMP activator forskolin, the ROCK pathway inhibitor Y‐27632, as well as specific neurogenic small molecules such as ISX‐9 [89–91].
⑤ Substrate/surface coating. In neural regeneration research involving hDPSCs, surface coating serves as a key strategy to regulate cell adhesion, proliferation, and directed differentiation, thereby directly impacting neural differentiation efficiency and the formation of functional neural structures [74, 92]. Neural differentiation induction experiments of hDPSCs are typically conducted in thermoplastic styrene (TPS) culture dishes. Gao et al. attempted to improve neural induction efficiency by enhancing surface coatings. They found that poly‐L‐ornithine‐coated thermoplastic polystyrene plates (TPS‐PLO) and plates coated with poly‐L‐ornithine and poly‐N‐isopropylacrylamide‐co‐butyl acrylate (TPS‐PLO‐PN) could support stable proliferation of hDPSCs and promote high expression of neuronal markers such as TUBB3 and Nestin [74]. hDPSCs seeded on poly‐L‐lysine‐coated plates can be induced into motor neurons, expressing neuron‐related markers such as HB9 and ISL LIM homeobox 1 (Islet‐1) [93]. In addition, type IV collagen, type I collagen, gelatin, polyornithine/fibronectin, and chitosan (CS) have all been used as surface coatings to enhance neural differentiation efficiency [94].
⑥ Neurosphere culture method: Neurospheres are formed by colonizing cells isolated from the CNS to form floating spheres [95]. The results show that sphere formation technology can better simulate in vivo cell growth, promote self‐renewal, prevent cell differentiation, and maintain the original characteristics of cells [96–98]. To date, a variety of methods have been used to induce neurosphere formation, including culture environment modification, surface modification, and culture dynamics. The most commonly used culture medium is serum‐free DMEM/F‐12, neurobasal medium and some commercial medium [82, 99, 100]. The choice of culture medium is critical for optimal neurosphere formation and regeneration. Spheroid formation on different surfaces can significantly affect neurosphere formation; examples of different surfaces used include plastic, glass, coated surfaces, and low attachment plates [101, 102]. In addition, Su et al. [103] tested the formation of spheres using dynamic or static culture methods; the medium flow was regulated by a peristaltic pump and set at 0.8 mL/min. In the dynamic culture environment, cells began to aggregate into spheres after 3 days, and the number of spheres increased over time.

The efficiency of neuronal differentiation of hDPSCs can be validated by assessing the expression of neuronal markers. Common approaches include immunofluorescence staining or qRT‐PCR to detect the expression of markers such as Nestin, MAP2, TUBB3, and glial fibrillary acidic protein (GFAP) [68]. However, because dental‐derived stem cells originate from the neural crest and can inherently express certain neuronal markers, additional electrophysiological characterization is required for proper identification. Arthur et al. [66] demonstrated the presence of voltage‐dependent sodium (Na^+^) influx in differentiated hDPSCs, although they did not observe individual PAs. Subsequently, Gervois et al. [68] successfully reported depolarization events resembling APs following hDPSCs neural differentiation. To date, however, no studies have shown that neural‐induced dental‐derived stem cells can establish functional synaptic contacts in co‐culture systems, indicating that current protocols for hDPSCs neuronal differentiation remain to be optimized.

hDPSCs can be driven toward neuron‐like phenotypes, but their degree of functional (synaptic and electrophysiological) maturity varies strongly with protocol and is generally partial compared with primary or long‑matured pluripotent stem cell–derived neurons. Differentiated hDPSCs express voltage‑gated Na^+^ and K^+^ currents (TTX‐ and TEA‐sensitive), with only a subset capable of firing a single AP, indicating immature excitability [68]. Optimization with retinoic acid and KCl pulses produced hDPSC‐derived neurons with robust voltage‐gated Na^+^/K^+^ currents, spontaneous activity, and repetitive AP trains with full baseline recovery, demonstrating substantially more mature excitability [104]. Across oral stem cells (OSCs), only specific neurosphere‐mediated protocols gave AP‑firing cells. 8% of hDPSC‐derived neurons fired APs, and no spontaneous postsynaptic events were detected, consistent with immature networks [105]. hDPSC‐derived neurons upregulate presynaptic markers (Synapsin I, Synaptophysin, vGLUT2) and postsynaptic scaffolds/receptors (PSD95, Gephyrin, glutamatergic and GABAergic receptor subunits), indicating synaptogenic machinery. Despite this, most studies report no or very rare spontaneous EPSCs/IPSCs, suggesting synapses are structurally present but functionally immature as networks [105]. hiPSC/hESC‐derived neurons can reach much higher maturity, with nearly all cells firing sustained AP trains and ~70%–75% showing spontaneous synaptic activity after optimized, weeks‐to‐months‐long protocols [106, 107]. Thus, relative to hiPSC/hESC models, hDPSC‐derived neurons currently resemble early embryonic/immature sensory or peripheral‐like neurons with partial synaptic and electrophysiological maturation [68, 104].

### 2.2. Paracrine Effects

Nosrat et al. proposed the neurotrophic effects of hDPSCs. Later, studies have suggested that the advantages of stem cell transplantation may be attributed to a paracrine effect rather than just the replacement of damaged cells at the injury site [46]. Its mechanisms of action encompass multiple aspects, including neuroprotection, immunomodulation, anti‐inflammatory effect, antiapoptotic effect, and angiogenesis. It is noteworthy that paracrine effects often occur concurrently with the process of cell differentiation into neurons and the replacement of damaged tissue, and the two cannot be completely separated.

① Neuroprotection. hDPSCs secrete neurotrophic factors that play important roles in neurogenesis, neural maintenance, and repair. They secrete NGF, BDNF, NT‐3, and GDNF to promote axonal growth [83]. hDPSCs are capable of neural differentiation. After neural induction, they can express neurotrophic proteins such as NGF, BDNF, and GDNF, activating ERK, AKT, Wnt pathways, and promoting the proliferation of neural‐induced hDPSCs [108]. This provides an effective and long‐term treatment for peripheral nerve injuries, facilitating functional recovery and anatomical repair [109].
② Immunomodulation and anti‐inflammatory effect. The secretome of hDPSCs exerts significant immunomodulatory and anti‐inflammatory effects through multiple mechanisms. Dental pulp stem cell‐conditioned medium (CM) and extracellular vesicles (EVs) are rich in immunomodulatory factors, which can regulate inflammatory responses and enhance the therapeutic efficacy for neurological disorders. hDPSCs secrete anti‐inflammatory cytokines such as IL‐6, IL‐10, and TGF‐β1, and suppress the expression of pro‐inflammatory cytokines including IL‐1β, IFN‐γ, IL‐2, IL‐12, and TNF‐α [110]. The secretome of hDPSCs inhibits the activation of microglia and promotes the polarization of macrophages from the pro‐inflammatory M1 phenotype to the anti‐inflammatory M2 phenotype [111]. The immunomodulatory and anti‐inflammatory effects of hDPSCs improve the neural microenvironment, reduce inflammation, and enhance tissue regeneration, highlighting the therapeutic potential of their secretome in neural repair [112].
③ Antiapoptotic effect. The secretome of hDPSCs exhibits significant anti‐apoptotic effects during neural differentiation. This is primarily achieved by upregulating anti‐apoptotic proteins B‐cell lymphoma 2 (Bcl‐2) and downregulating pro‐apoptotic factors (including Bcl‐2‐associated X protein Bax, p53, and Caspase‐3), thereby protecting neural cells [112, 113]. The secretome of hDPSCs contains high levels of neurotrophic factors such as VEGF, Fractalkine, and granulocyte‐macrophage colony‐stimulating factor (GM‐CSF), which can reduce cytotoxicity and apoptosis induced by β‐amyloid (Aβ). This effect is achieved by enhancing cell viability through the stimulation of endogenous survival factor Bcl‐2 and the downregulation of the pro‐apoptotic regulator Bax [114]. In a rat spinal cord injury (SCI) model, transplantation of hydrogel loaded with β‐FGF and dental pulp stem cells can inhibit the expression of Bax and Caspase‐3 proteins, thereby suppressing apoptosis [115].
④ Angiogenesis. Growth factors BDNF, NGF, and VEGF can promote endothelial cell migration and angiogenesis through paracrine effects of hDPSCs, while enhancing nerve cell differentiation and axon growth [87, 112]. hDPSCs have been shown to express angiogenic markers like VEGF, platelet‐derived growth factor subunit A (PDGFA), and angiopoietin‐1 (ANG‐1), which are essential for blood vessel formation. This angiogenic potential is crucial for supporting nerve regeneration by ensuring adequate blood supply [116, 117]. The multiple bioactive factors secreted by hDPSCs can not only promote the formation of new blood vessels but also enhance neural differentiation and neural repair capabilities.

### 2.3. The Synergistic Interplay Between Stem Cell Therapies and Biomaterial Scaffolds

A key challenge in stem cell‐based nerve repair is how to accurately and effectively deliver hDPSCs to the site of neural injury and establish a microenvironment conducive to their differentiation. Selecting an appropriate biomaterial scaffold can facilitate the targeted delivery of hDPSCs and promote their migration and differentiation toward neural lineages. Natural polymers, synthetic polymers, graphene, and hydrogels have been widely used to support and guide the neural differentiation of stem cells [94, 118].

CS scaffolds, owing to their excellent biocompatibility and porous structure, have become a commonly used carrier for the neural differentiation of hDPSCs. Research indicates that a pore size of 268.79 ± 13.25 μm is optimal for nerve growth and migration. This scaffold synergises with β‐FGF to enhance expression of neural markers such as GFAP, S100β, and TUBB3 by activating the ERK/p‐ERK signaling pathway [119]. To address the shortcomings of insufficient hydrophilicity and thermal stability, the study enhanced physical properties by incorporating organic montmorillonite (OMMT) or improved hydrophilicity through the addition of polyvinyl alcohol (PVA). A 5% OMMT composite ratio significantly induced neural differentiation [120]. CS tubes loaded with hDPSCs and stem cell factor (SCF) implanted into a rabbit facial nerve defect model demonstrated significantly more myelinated nerve fibers, greater myelin thickness, and larger nerve diameters compared to the CS‐only group after 12 weeks. Furthermore, CD31 protein expression approached levels seen in the autologous nerve graft group, achieving synergistic nerve‐vascular regeneration [121].

Polymer scaffolds demonstrate outstanding performance in hDPSC neural differentiation due to their controllable structural design advantages. The pillar spacing width of 3D‐printed polylactic acid scaffolds (3DP‐PLAS) is a key regulatory factor. Research confirms that a 150 μm gap more readily induces cell orientation than a 200 μm gap. Following alcohol immersion or poly‐L‐lysine coating treatment, cell adhesion significantly improves, successfully inducing expression of neural markers such as Nestin and MAP2 [122]. Reduced graphene oxide (RGO)‐polycaprolactone (PCL) composite nanofiber scaffolds combine structural support with electrical conductivity advantages. An optimal ratio of 0.1% RGO concentration promotes the expression of neural markers such as TUBB3 and NeuN. Among these, aligned nanofibers (AFs) facilitate the construction of unidirectional neural networks, while random nanofibers (RFs) are suitable for forming multidirectional neural connections [123].

Hydrogel scaffolds demonstrate outstanding performance in growth factor loading and cell delivery due to their high water content and biomimetic properties. Gelatin methacrylate (GelMA) hydrogels are a research hotspot. At a 10% GelMA concentration combined with bFGF, they exhibit ideal biocompatibility, degradability, and swelling rate. Their porous structure promotes nutrient exchange and sustained release of growth factors, achieving a cumulative β‐FGF release rate of 52.67 ± 2.08% within 28 days. When incorporated into cellulose/soybean protein composite membrane (CSM) tubes to form CSM‐GFD conduits, these materials demonstrated comparable outcomes in a rat 15 mm sciatic nerve defect model. After 12 weeks, the number of myelinated fibers in regenerated nerve tissue showed no significant difference compared to the autologous nerve graft group. Sciatic Nerve Function Index (SFI) recovery was equivalent, with the regenerated nerve tissue primarily derived from the direct differentiation of exogenous hDPSCs [124]. Type I collagen hydrogels can induce hDPSCs to differentiate into Schwann cell‐like phenotypes, promoting axonal growth and extension by providing cellular alignment guidance [125]. Digital light processing (DLP) 3D‐printed GelMA microspheres loaded with hDPSCs exhibited an elastic modulus of 34.33 ± 6.00 kPa after 5 days of incubation, closely matching that of natural human dental pulp (32.60 ± 9.48 kPa). Their surface features a uniform microporous structure, facilitating nutrient exchange and metabolic waste removal. In vitro, these microspheres significantly enhanced expression levels of the neural markers GAP43 and MAP2. In a rat SCI model, after 8 weeks, both motor function scores and tilt plane test results outperformed those of the cell aggregate and cell suspension groups. The regenerated neural tissue exhibited the highest intensity of GAP43 and MAP2 positive expression [118].

The core design elements of neural differentiation scaffolds for dental pulp stem cells include appropriate pore size, elastic modulus matching natural tissue, controllable degradation rate, excellent cell compatibility, and synergistic delivery capability with growth factors or secretome. Current research focuses on optimizing CS‐based scaffold modifications, precisely controlling the structure of 3D‐printed polymer scaffolds, and functionalizing GelMA hydrogels and microspheres. Future efforts should focus on further optimizing scaffold physicochemical properties and functional compatibility, conducting more clinical translation studies, and providing efficient tissue engineering solutions for nerve injury repair and neurodegenerative disease treatment.

### 2.4. Crosstalk and Hierarchy of the Key Signaling Pathways

The neural differentiation of hDPSCs is not governed by a single pathway, but rather by a network of multiple pathways, transcription factors, and microRNAs. Crosstalk and hierarchy exist among Wnt, MAPK/ERK, Hippo, ROS, Notch pathways, and microRNAs.

The Wnt/β‐catenin pathway promotes neural differentiation. WNT3A and activated β‐catenin increase during neural induction of hDPSCs. Neural markers are upregulated. Non‐canonical Wnt/Ca^2+^ signaling inhibits hDPSCs neural differentiation while simultaneously downregulating Wnt/β‐catenin (canonical Wnt) pathway activity. When inhibitory Wnt5a‐Ca^2+^ signaling decreases, β‐catenin signaling and mature neural markers express increase [126]. Canonical Wnt and Notch synergistically maintain the expression of neural crest and core pluripotency factors of hDPSCs. Inhibiting Notch concomitantly silences canonical Wnt, reducing multipotent differentiation capacity [127]. ERK/MAPK activity is essential for hDPSC differentiation into cholinergic sensory neurons; the ERK1/2 inhibitor U0126 significantly impaired hDPSC neuronal differentiation [62]. Recombinant PrP activates ERK1/2 and Akt, inducing upregulation of TUBB3, NF‐H, and GAP43. Silencing endogenous PrP or lipid rafts blocks ERK/Akt activation and neural differentiation, demonstrating that the lipid raft‐PrP‐ERK/Akt cascade forms a pro‐neural axis [128]. Activation of the Hippo pathway, manifested by YAP phosphorylation and nuclear exclusion, promotes dopaminergic (DAergic)‐like neuronal differentiation while inhibiting proliferation and apoptosis when combined with melatonin treatment. This demonstrates the gating role of Hippo in the “exit cell cycle‐enter neural lineage” transition [129]. Armadillo repeat containing X‐linked 3 (ARMCX3) is upregulated in inflammatory environments, enhancing ROS and inhibiting neural differentiation; ARMCX3 knockdown suppresses ROS, restoring/enhancing neural differentiation and reducing inflammatory factor expression. ROS‐ARMCX3 negatively regulates axonal differentiation and couples with inflammatory signaling [130]. Overexpression of miR‐22‐3p inhibits neural progenitor‐like differentiation and promotes apoptosis; inhibiting miR‐22‐3p enhances Nestin expression and proliferation, facilitating acquisition of more neural progenitors [131]. EphrinB2 activation inhibits the expression of TUBB3, NCAM, nestin, and neurogenin 2 (NGN2). Blocking EphB4 promotes the expression of neural markers MAP2, Musashi1, NGN2, and neuron‐specific enolase [132].

## 3. CNS Injuries

### 3.1. SCI

SCI is a serious neurological disorder. It causes loss of motor function, pain, and autonomic dysfunction [133]. Current treatments focus on surgery and drugs to promote the recovery of nerve function. However, there are no effective therapies for irreversible neural injury. hDPSCs show great potential in SCI treatment due to their anti‐inflammatory, antiapoptotic, and axon regeneration‐promoting properties (Figure 2A) [134].

Figure 2Therapeutic effects of hDPSCs on central nervous system injuries. (A) In spinal cord injury, hDPSCs reduce inflammation, apoptosis, and promote axon regeneration. (B) In stroke, hDPSCs secrete neurotrophic factors, reduce infarct volume, and suppress neuroinflammation. (C) In Parkinson’s disease, hDPSCs differentiate into dopaminergic‐like neurons and modulate inflammatory responses. (D) In Alzheimer’s disease, hDPSCs have neuroprotective effects, cognitive improvement, and prolongation of dendrites. This schematic was generated using Nano Banana2 and manually verified for accuracy by the authors.

**Figure:**
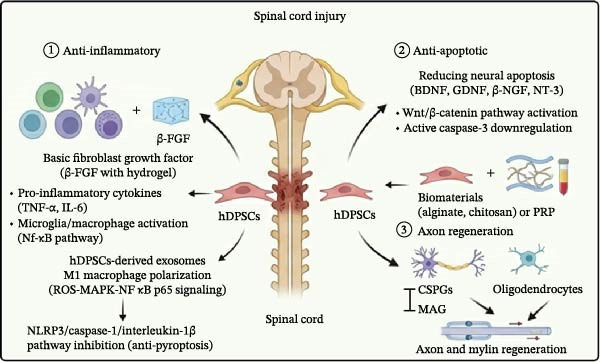
(A)

**Figure:**
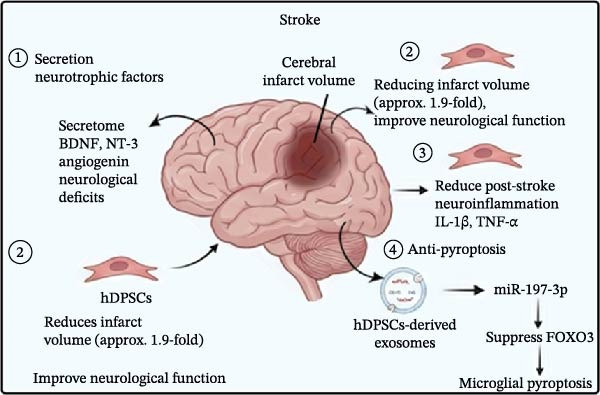
(B)

**Figure:**
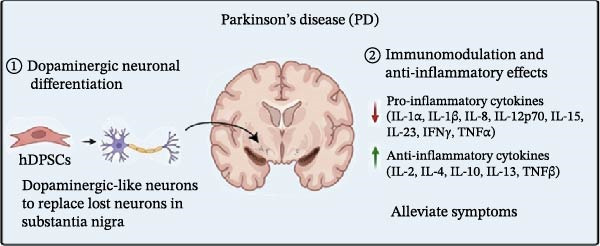
(C)

**Figure:**
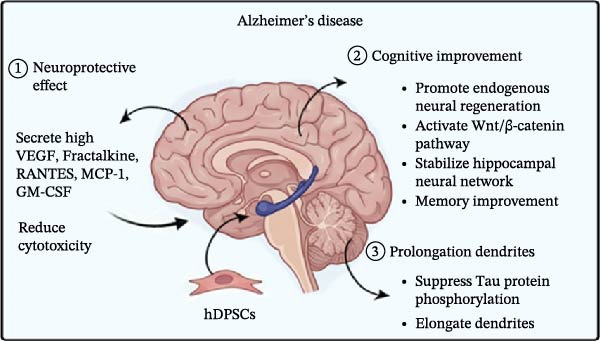
(D)

① Anti‐inflammatory: After SCI, the inflammatory response spreads the damage and induces apoptosis, leading to further loss of neurons and axons and impeding spontaneous regeneration and functional recovery [135]. hDPSCs modulate immune cell activity, including T cells, B cells, dendritic cells, and NK cells, to inhibit proinflammatory processes and enhance anti‐inflammatory responses [136–138]. hDPSCs exert neuroprotective effects in SCI by modulating the local inflammatory milieu, suppressing pro‐inflammatory cytokines (TNF‐α and IL‐6). The combination ofβ‐FGF with hDPSCs in a thermosensitive hydrogel inhibits the activation of microglia/macrophages. β‐FGF‐hDPSCs inhibit TNF‐α and IL‐6 release by suppressing the NF‐κB pathway, thereby alleviating tissue inflammation in SCI and promoting neural repair [111]. In a SCI model, hDPSCs‐derived exosomes suppress the polarization of macrophages toward the pro‐inflammatory M1 phenotype through the ROS‐MAPK‐NFκB p65 signaling pathway, thereby reducing IL‐1β and TNF‐α‐induced cellular damage [139]. hDPSCs have been shown to inhibit microglial pyroptosis, a form of programed cell death associated with inflammation, by suppressing the NLRP3/caspase‐1/interleukin‐1β pathway. This inhibition leads to reduced inflammation and promotes neurological recovery in SCI models [140].
② Anti‐apoptotic: hDPSCs effectively reduce neuronal apoptosis after SCI and promote nerve regeneration and functional recovery through multiple mechanisms. hDPSCs promote motor function recovery and inhibit cell apoptosis after SCI by secreting high levels of neurotrophic factors such as BDNF, GDNF, β‐NGF, and NT‐3, activating the Wnt/β‐catenin signaling pathway, and downregulating the expression of active caspase‐3 [141]. Transplantation of hDPSCs significantly reduces apoptosis of neurons, astrocytes and oligodendrocytes in the SCI region, protects nerve fibers and myelin structures, and promotes functional recovery [142]. hDPSCs can reduce neuronal apoptosis and exert neuroprotective effects through the miR‐26a‐5p/PTEN/AKT pathway [143]. The combination of hDPSCs with biomaterials (e.g., alginate hydrogel, CS scaffolds) or platelet‐rich plasma (PRP) further enhances the antiapoptotic and neuroregenerative effects, significantly reduces the rate of neuronal apoptosis and improves motor function [144].
③ Axon regeneration: Multiple animal studies have shown significant improvement in motor and sensory function, increased axonal and myelin regeneration, and more complete organization of the damaged spinal cord following hDPSCs transplantation. hDPSCs can differentiate into mature neurons and oligodendrocytes at the site of injury, further promoting axon growth and spinal cord regeneration [142]. hDPSCs not only differentiate into neurons and oligodendrocytes but also inhibit a variety of axon growth inhibitory factors (e.g., chondroitin sulfate proteoglycans, CSPGs and myelin‐associated glycoprotein, MAG) and promote axon regeneration through a paracrine mechanism [142]. Conditioned medium from hDPSCs has been found to inhibit microglial pyroptosis by suppressing the NLRP3/caspase‐1/interleukin‐1β pathway. This inhibition supports axonal and myelin regeneration [140].

### 3.2. Stroke

hDPSCs significantly improved post‐stroke neurological function and reduced cerebral infarct volume. Specifically, hDPSCs work through several mechanisms (Figure 2B).

① Secretion neurotrophic factors: The secretome of hDPSCs, rich in trophic factors, has demonstrated short‐term benefits in reducing neurological deficits and infarct size in rat models of mild ischemic stroke (IS). This effect is attributed to the modulation of neurotrophic and angiogenic factors, such as BDNF, NT‐3, GDNF, and angiogenin, which are crucial for anti‐apoptosis, neuronal survival, and angiogenesis [145].
② Neuroprotective effects: hDPSCs exhibit notable neuroprotective effects in IS, significantly reducing infarct volume and improving neurological function. Studies report that hDPSCs transplantation decreases infarct volume by ~1.9‐fold compared to control groups. This improvement is evident within 1 day, peaking at 7 days, and maintaining partial protective effects for up to 2 weeks [146].
③ hDPSCs reduce post‐stroke neuroinflammation by downregulating the expression of pro‐inflammatory cytokines such as IL‐1β and TNF‐α. These cytokines are key contributors to neural damage following stroke, and their inhibition by hDPSCs helps create a more favorable environment for neural recovery [147, 148].
④ Anti‐pyroptosis: hDPSCs‐derived exosomes inhibit microglial pyroptosis by delivering miR‐197‐3p to suppress FOXO3 expression, thereby contributing to stroke therapy [149].

### 3.3. Parkinson’s Disease (PD)

hDPSCs influence the treatment of PD through multiple mechanisms.

① DAergic neuronal differentiation: hDPSCs have demonstrated the ability to differentiate into DAergic neurons, which are the primary type of neurons lost in PD. This differentiation is crucial for potentially replacing the lost neurons and restoring function in the substantia nigra, the brain region most affected by PD [150–152]. In vitro studies have demonstrated that hDPSCs can be induced to differentiate into DAergic neuron‐like cells in specific experimental conditions [153].
② Immunomodulation and anti‐inflammatory effects: Injuries can lead to inflammation, and neuroinflammation contributes to the death of DAergic neurons in the substantia nigra of PD patients. hDPSCs reduce pro‐inflammatory cytokines (IL‐1α, IL‐1β, IL‐8, IL‐12p70, IL‐15, IL‐23, IFNγ, and TNFα) and increase anti‐inflammatory cytokines (IL‐2, IL‐4, IL‐10, IL‐13, and TNFβ), thereby alleviating symptoms in PD models. This suggests that hDPSCs exert therapeutic effects through a dual mechanism of neuroregeneration and immunomodulation (Figure 2C) [154].

### 3.4. Alzheimer’s Disease (AD)

Alzheimer’s is a progressive neurodegenerative disease characterized by nerve cell dysfunction and a decrease in the number of neurons in the brain, leading to long‐term memory dysfunction and cognitive decline. With in‐depth research into the pathogenesis of AD, there is growing evidence that hDPSCs have the potential to ameliorate the condition (Figure 2D).

① Neuroprotective effect: hDPSCs secrete high levels of VEGF, Fractalkine, RANTES, MCP‐1, and GM‐CSF, which contribute to reduced cytotoxicity and apoptosis [114].
② Cognitive improvement: hDPSCs promote endogenous neural regeneration, particularly in the hippocampus, a region severely affected by AD. They activate the Wnt/β‐catenin pathway, which is crucial for stabilizing the hippocampal neural network and reversing memory deficits. In animal models, single transplantation of hDPSCs has resulted in significant improvements in cognitive function and neuropathological features. These benefits were observed both in the short term and sustained over several months [155].
③ Prolongation of dendrites: Wang et al. [156] found that hDPSCs suppress Tau protein phosphorylation and elongate dendrites.

### 3.5. Clinical Trials and Applications

Clinical translation of hDPSCs has achieved significant breakthroughs. In the field of neurodegenerative diseases, results from a Phase I open‐label clinical trial involving intravenous injection of immature hDPSCs (NestaCell HD) to treat Huntington’s disease (HD) patients showed no adverse reactions in subjects during 48 h of intensive care monitoring or within 15 days post‐infusion. During the 2‐year follow‐up period, no treatment‐related serious adverse events (SAEs) occurred. The few events considered treatment‐related were transient hair pigment changes or regrowth, with no clinically significant abnormal fluctuations in cytokine or lymphocyte levels observed. Regarding preliminary efficacy, 5 of 6 patients demonstrated improvement in the motor domain of the Unified HD Rating Scale (UHDRS) within 2 weeks of the first dose, with this improvement persisting for 6–9 months after the last dose. Furthermore, preliminary analysis suggests the treatment may have stabilized the decline in patients’ Total Motor Score (TMS) and Total Functional Capability (TFC) [157].

Based on favorable safety feedback from the Phase I trial, the subsequent Phase II multicenter, randomized, double‐blind, placebo‐controlled trial (NCT04219241) expanded the participant cohort to evaluate the therapeutic efficacy of repeated intravenous infusions of NestaCell in patients with early‐to‐mid‐stage HD. Using the UHDRS TMS and TFC as primary efficacy endpoints, this study aims to validate the long‐term potential of hDPSCs in slowing neurological decline, improving motor impairment, and stabilizing CNS neural networks through neuromodulatory effects. This advancement not only validates the clinical applicability of hDPSCs but also marks a pivotal transition in cell therapy for inherited neurodegenerative diseases—shifting from “safety evaluation” to “confirmatory efficacy assessment [158].”

## 4. Peripheral Nerve Injury

### 4.1. Sciatic Nerve Injury

Sciatic nerve injury is a common type of peripheral nerve injury. The mechanism of hDPSCs in sciatic nerve repair is mainly reflected in the following aspects (Figure 3A):

Figure 3Therapeutic effects of hDPSC‐derived secretome in peripheral nerve injury. (A) In sciatic nerve injury, hDPSC‐derived exosomes promote myelin regeneration and enhance Schwann cell function. (B) In facial nerve injury, hDPSCs facilitate neural differentiation and, when combined with scaffolds (e.g., chitosan, graphene), enhance nerve repair and functional recovery. This schematic was generated using Nano Banana2 and manually verified for accuracy by the authors.

**Figure:**
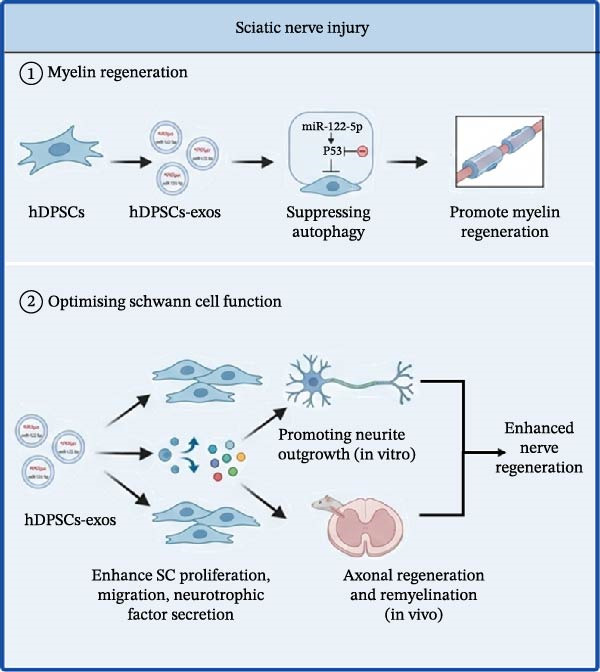
(A)

**Figure:**
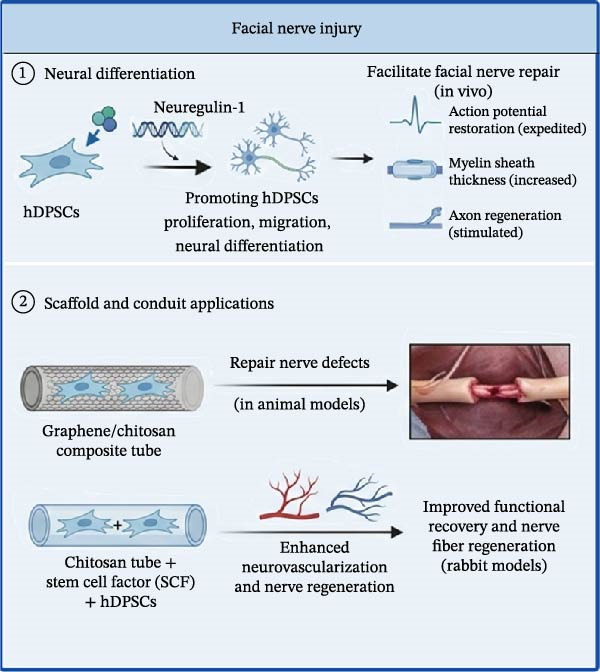
(B)

① Myelin regeneration：hDPSC‐exos could promote the regeneration of the myelin sheath through suppressing the autophagy in Schwann cells caused by sciatic nerve injury via miR‐122‐5p/P53 pathway [159].
② Optimizing Schwann cell function: hDPSCs‐exos have been shown to enhance Schwann cell proliferation, migration, and neurotrophic factor secretion, thereby promoting neurite outgrowth in vitro. In vivo studies using rat sciatic nerve injury models revealed that hDPSCs‐exos administration leads to marked improvements in axonal regeneration and remyelination, highlighting their therapeutic potential for nerve regeneration [160].

### 4.2. Facial Nerve Injury

① Neural differentiation: Specific actions and mechanisms of hDPSCs to promote facial nerve injury repair include neural differentiation. Neuregulin‐1 has been shown to enhance the regenerative capacity of hDPSCs by promoting their proliferation, migration, and neural differentiation. This gene facilitates the repair of facial nerve injuries by expediting AP restoration, increasing myelin sheath thickness, and stimulating axon regeneration in vivo [161].
② Scaffold and conduit applications. The combination of graphene/CS tubes with hDPSCs has been investigated for its potential to repair facial nerve injuries. This approach leverages the favorable physical and chemical properties of the composite tubes and the regenerative capabilities of hDPSCs, resulting in enhanced repair of nerve defects in animal models [162]. CS tubes inoculated with SCF and hDPSCs have shown promise in enhancing neurovascularization and nerve regeneration. SCF promotes the biological activity and neural differentiation of hDPSCs, leading to improved functional recovery, and nerve fiber regeneration in rabbit models (Figure 3B) [121].

As previously described, hDPSCs promote neural repair through the interaction of multiple bioactive factors, many of which exhibit multifunctionality—acting via diverse mechanisms across different disease models. To clearly illustrate these molecular interactions, Table 2 summarizes key molecules/factors, their multidimensional mechanisms of action, and research domains targeting specific types of neural injury.

**Table 2:** Pleiotropic factors and therapeutic mechanisms of hDPSCs in neural repair.

Molecule/factor	Mechanism(s) of action	Diseases
β‐FGF	Anti‐inflammatory: β‐FGF inhibit of pro‐inflammatory factors IL‐6 and TNF‐α	SCI [111]
BDNF	Anti‐apoptotic: Activates Wnt/β‐catenin to inhibit caspase‐3; promotes neuritogenesis, axonal growth	SCI [141]
	Neurotrophic function: BDNF activates the intracellular PI3K/Akt pathway by binding to the specific receptor TrkB on the surface of neurons	Stroke [145, 163]
NT‐3	Anti‐apoptotic: Activates Wnt/β‐catenin to inhibit caspase‐3; promotes neuritogenesis, axonal growth	SCI [141]
	Neurotrophic Support: Elevate the neuroplasticity of spared corticospinal axons	Stroke [164]
Angiogenin	Neurotrophic Support: Trigger cell growth, endothelial cell proliferation, migration, and cell invasion	Stroke [145]
GDNF	Anti‐apoptotic: In conjunction with other factors, creates a conducive and favorable microenvironment, promoting the survival of transplanted cells	SCI [141]
	Neurotrophic Support: Promote cell development, neuronal plasticity, synaptic remodeling, neuronal differentiation, and cell survival	Stroke [145]
VEGF, Fractalkine	Neuroprotective effect: hDPSCs secrete VEGF and synergistic factors to collectively improve the adverse microenvironment, reducing neuronal cell death and cytotoxic damage	AD [114]
miR‐26a‐5p	Anti‐apoptotic: miR‐26a‐5p downregulates Bax and Caspase‐3 via the PTEN/AKT pathway and upregulation of Bcl‐2 to promote apoptosis	SCI [143]
miR‐197‐3p	Anti‐pyroptosis: Exosomally delivered to suppress FOXO3 expression, thereby inhibiting microglial inflammatory death	Stroke [149]
miR‐122‐5p	Myelin regeneration：Suppressing the autophagy in Schwann cells via miR‐122‐5p/P53 pathway	Sciatic nerve injury [159]
Neuregulin‐1	Premote regeneration: Facilitates action potential restoration and increases myelin thickness while stimulating axonal growth	Facial nerve injury [161]

Abbreviations: AD, Alzheimer’s disease; Bax, Bcl‐2‐associated X protein; Bcl‐2, B‐cell lymphoma 2; BDNF, Brain‐derived neurotrophic factor; β‐FGF, Basic fibroblast growth factor; FOXO3, Forkhead box O3; GDNF, Glial cell line‐derived neurotrophic factor; hDP‐SCs, Human dental pulp stem cells; IL‐6, Interleukin‐6; miR, MicroRNA; NT‐3, Neurotrophin‐3; PI3K/Akt, Phosphoinositide 3‐kinase/Protein kinase B; P53, Tumor protein p53; PTEN, Phosphatase and tensin homolog; SAH, Subarachnoid hemorrhage; SCI, Spinal cord injury; TNF‐α, Tumor necrosis factor‐alpha; TrkB, Tropomyosin receptor kinase B; VEGF, Vascular endothelial growth factor; Wnt/β‐catenin, Wingless‐related integration site/beta‐catenin.

## 5. Balance Between Cell Replacement and Paracrine Mechanisms Across Neurological Disorders

Research evidence indicates that the therapeutic mechanisms of hDPSCs for neurological disorders involve complex interactions between direct cell replacement and paracrine effects. The relative importance of each mechanism varies significantly depending on the disease context and regenerative potential.

During the acute phase of SCI, inflammatory storms, secondary injuries, and glial scar formation occur. Most directly transplanted cells exhibit low survival rates, with an extremely low proportion achieving true integration into the injured tissue [165]. The therapeutic improvements from stem cell treatment primarily stem from anti‐inflammatory effects, anti‐apoptotic actions, promotion of axonal regeneration, suppression of glial scarring, and secretion of neurotrophic factors. Therefore, functional improvement in SCI is primarily attributed to paracrine effects rather than stable neuronal/glial cell replacement [166]. Furthermore, literature indicates that strategies utilizing only exosomes or secretomes can replicate most of these benefits, highlighting the advantages of paracrine mechanisms [167].

For stroke, current evidence indicates that therapeutic effects are predominantly driven by paracrine mechanisms rather than direct cell replacement. Preclinical studies consistently demonstrate that following intravenous administration, the majority of transplanted stem cells are trapped in peripheral organs with minimal engraftment and extremely low structural integration within the brain, arguing against significant neuronal replacement as a primary mode of action [168]. Functional recovery in many animal models often occurs earlier than the timeframe required for neuronal differentiation and circuit integration, further supporting a paracrine‐dominated mechanism [169]. Mechanistically, stem cells and their EVs exert anti‐inflammatory, immunomodulatory, pro‐angiogenic, and neuroprotective effects, including modulation of microglial activation, secretion of trophic factors such as VEGF, BDNF, and β‐FGF, enhancement of angiogenesis and blood–brain barrier preservation, activation of endogenous progenitor cells, and promotion of synaptic remodeling [170]. These collective bystander actions facilitate neurologic improvement in IS models without substantial evidence of direct neuronal replacement.

Research indicates that the therapeutic effects of hDPSCs in PD primarily stem from paracrine‐mediated neurotrophic support and immunomodulation, including the secretion of factors such as BDNF and GDNF to protect residual DAergic neurons and suppress neuroinflammation [150]. Although hDPSCs can differentiate into dopamine‐like neurons expressing markers like tyrosine hydroxylase (TH) and dopamine transporter (DAT) under specific induction conditions, and partially restore dopamine levels and motor function in animal models, evidence for their long‐term survival and functional integration within the nigrostriatal pathway remains limited [153]. Consequently, most studies suggest their efficacy primarily relies on paracrine neuroprotection, accompanied by some cell replacement potential. Future therapeutic strategies may increasingly favor a combined mechanism emphasizing paracrine protection with limited neural replacement as a secondary component [150]. The therapeutic role of hDPSCs in AD primarily relies on paracrine‐mediated neuroprotection and microenvironment regulation, including anti‐inflammatory and antioxidant effects, promotion of endogenous neurogenesis, and facilitation of Aβ clearance via enzymes such as NEP [114]. Multiple neurotrophic factors and cytokines secreted by hDPSCs can alleviate neuroinflammation, inhibit neuronal apoptosis, and improve cognitive function. Although hDPSCs can differentiate into neuron‐like cells under specific conditions and express neural markers such as NeuN and DCX, suggesting potential for cell replacement, evidence for their long‐term survival and functional integration within the brain remains limited [171]. Consequently, the prevailing view is that the mechanism of hDPSC therapy for AD primarily involves paracrine neuroprotection, with neural replacement playing a secondary role.

For PNIs, stem cells promote regeneration chiefly by paracrine support of endogenous Schwann cells, macrophage polarization, angiogenesis, and axonal growth, with differentiation into Schwann‑like cells as an adjunct rather than the primary driver [172].

## 6. Challenges and Optimization Strategies

### 6.1. Regulation of Differentiation Propensity

Although hDPSCs possess neurogenic differentiation potential, they naturally tend to differentiate toward osteogenic or odontogenic lineages, posing a major challenge for neural regeneration applications. To enhance their neuro‐directed differentiation capacity, researchers have implemented regulatory optimisations at multiple levels. At the genetic level, overexpression of Oct4 enhances hDPSCs’ stemness and activates early neural markers (SOX1 and SOX2), significantly improving neural progenitor differentiation efficiency [89]. KLF2 has been demonstrated to regulate autophagy and mitochondrial function through the Wnt5a signaling pathway, while simultaneously promoting neural differentiation and inhibiting non‐neural lineage commitment [173]. Inhibition of miR‐22‐3p enhances neural differentiation and proliferation of hDPSCs, while its overexpression disrupts synaptic function and lipid metabolism, leading to apoptosis [131]. Furthermore, inhibition of the heat shock protein HSP27 has been shown to promote neural differentiation [174]. Exogenous microenvironmental modulation also plays a significant role. Photobiomodulation (PBM) can noninvasively enhance neural differentiation efficiency [165]. Together, these strategies provide feasible avenues for achieving efficient and directed neural differentiation of dental‐derived stem cells, laying the groundwork for their clinical translation in neural tissue engineering and nerve injury repair.

### 6.2. Delivery Strategies

To enhance the application efficiency of hDPSCs in neural tissue engineering, recent research has increasingly focused on optimizing stem cell delivery strategies, particularly the combined use of three‐dimensional (3D) culture systems and biomaterial scaffolds. On one hand, the 3D culture environment better maintains cellular organization and intercellular communication, which improves neurogenic differentiation capacity [72]. On the other hand, biomaterials such as gelatin sponges, CS, decellularised extracellular matrix, and conductive hydrogels can mimic the mechanical tension, conductivity, and surface architecture of neural matrices, thereby enhancing hDPSCs adhesion, survival, and induction efficiency [94, 141, 175]. For example, Fakhr et al. used PVA/CS nanofibers to construct a biomimetic conductive scaffold that successfully promoted axon‐like extension and neural marker gene expression in hDPSCs [176]. Similarly, Mansouri et al. [177] employed graphene‐based scaffolds to enhance neuronal‐like differentiation and neural network formation. Furthermore, Khatami et al. [94] highlighted in their review that polymeric scaffolds, CS scaffolds, hydrogel scaffolds, and decellularised extracellular matrix can serve as effective delivery carriers, improving the cellular microenvironment and increasing neural lineage induction efficiency. Biological scaffolds, nanofibrous materials, or conductive hydrogels can induce hDPSCs to differentiate along the neural lineage by mimicking the neural tissue microenvironment through mechanical, electrical, and surface structural cues [74, 176, 178]. These strategies have significantly improved the biocompatibility, viability, and neuroinduction efficiency of hDPSCs, providing a solid foundation for their clinical translation in neural regeneration.

### 6.3. Clinical Trials and Clinical Translation Limitations

The clinical translation of hDPSCs has moved from theoretical potential to early‐phase human trials, offering a unique perspective on their therapeutic efficacy. Recent breakthroughs have focused on neurodegenerative conditions and acute injuries.

① HD: A first‐in‐human, open‐label Phase I clinical trial evaluated the safety of NestaCell (immature hDPSCs). The breakthrough of this study lies in successfully utilizing hDPSCs extracted from healthy children’s shed baby teeth. These cells demonstrated superior axon growth promotion and enhanced neurological development compared to conventional MSCs, along with lower immunogenicity. In Phase I clinical trials, they exhibited exceptional safety and potential for functional improvement. The core limitation for clinical translation centers on the context‐dependent and dose‐dependent nature of stem cell immunomodulation. Fluctuations in lymphocyte counts observed among different subjects post‐infusion reflect the uncertainty of stem cells within complex in vivo environments, complicating efforts toward standardized treatment. Although genetic analysis indicates the lung cancer in this case was unrelated to the stem cells, the long‐term potential tumorigenic risk of stem cell therapy remains a critical focus requiring heightened attention from regulatory bodies and clinical translators [157]. HD‐Phase II Trial: Building on the Phase I safety data, a randomized, double‐blind, placebo‐controlled Phase II trial was conducted with 35 patients. This study demonstrates the safety and preliminary efficacy of NestaCell in treating HD. The study found that through regular intravenous infusions, patients demonstrated significant improvements in motor function (TMS scores) and functional capacity (TFC scores) compared to the placebo group, without requiring immunosuppression. Despite limitations including a small sample size and insufficient imaging evidence, this research pioneers a non‐invasive stem cell repair pathway for HD [158].
② Acute IS: This study represents the first randomized, double‐blind, placebo‐controlled, multicenter Phase I/II clinical trial of JTR‐161 (allogeneic hDPSCs) in acute IS. It innovatively employs a single intravenous infusion within 48 h of symptom onset and evaluates efficacy using a stringent “excellent outcome” composite endpoint (mRS ≤ 1, NIHSS ≤ 1, BI ≥ 95) to assess efficacy, propelling hDPSCs from animal studies into formal clinical validation. As an exploratory early‐stage trial, it features a small sample size, Japanese‐only population, and a primary focus on safety and dose exploration, with efficacy evidence still in the validation phase. Concurrent standard reperfusion therapy may complicate efficacy assessment, necessitating larger Phase III studies to confirm genuine clinical benefit [179].

As hDPSCs advance toward clinical applications, donor variability, genomic stability, GMP manufacturing constraints, limited synaptic integration, immunogenicity, and tumorigenic risk have emerged as critical factors that compromise the quality and reproducibility of cell products.

① Donor variability. Donor variability exerts a profound impact on hDPSC biological properties. Multiple studies indicate that with increasing donor age, hDPSCs exhibit diminished proliferative capacity and heightened apoptosis, though their overall differentiation potential remains largely preserved. Younger donors (≤35 years old) demonstrate superior maintenance of high proliferative capacity during long‐term passage [180]. In fact, the functional performance of hDPSCs is jointly constrained by a combination of donor‐related factors including age, telomere length, and general health status. Typically, increased donor age, telomere shortening, or extended in vitro passage number all lead to a significant decline in hDPSC proliferation rate, telomere activity, and osteogenic/odontogenic differentiation potential. This high degree of heterogeneity across individual donors and clonal cell samples poses a major challenge to ensuring stable and consistent regenerative outcomes in clinical translation. Consequently, current research emphasizes the necessity of precisely identifying and selecting high‐performance cell subpopulations through standardized isolation techniques and single‐cell sequencing to mitigate the adverse effects of donor heterogeneity on therapeutic efficacy [181].
② Genomic stability. Genomic stability, which is closely linked to cryopreservation protocols, also requires stringent regulation for clinical‐grade hDPSC products. Cryopreservation parameters including cooling rate, cryoprotectant type and concentration, and storage duration can directly influence post‐thaw cell viability, phenotypic stability, and potentially genomic integrity [182]. Standardization of these cryopreservation procedures is therefore an essential prerequisite to safeguarding the genomic stability of hDPSCs and ensuring the reliability of clinical cell products.
③ GMP manufacturing constraints. Although hDPSCs have demonstrated promising regenerative potential in animal studies and limited clinical trials, their clinical translation remains severely constrained by GMP‐related requirements. Core bottlenecks are concentrated in two key areas: process quality and regulatory frameworks. Regarding process quality, traditional culture systems containing fetal bovine serum (FBS) pose safety risks. Existing serum‐free, xenogeneic‐free systems still face challenges such as long‐term passage‐induced phenotypic changes. Additionally, risks of senescence and genetic instability during passage expansion, coupled with GMP‐grade requirements for raw materials and equipment, further increase preparation complexity and costs [183]. Regarding regulatory frameworks, hDPSCs are classified as Advanced Therapy Medicinal Products, subjecting them to stringent GMP and quality system requirements. Compounding these challenges are the absence of standardized preparation protocols, logistical complexities unique to dental applications, and the imbalance between high production/testing costs and the benefits of alternative treatments for oral diseases—all of which further impede clinical translation [184]. Current primary strategies to address these challenges include: developing standardized, serum‐free/xeno‐free GMP culture systems with controlled passage limits to balance cell yield and quality; establishing allogeneic hDPSC/HLA‐typed cell banks to distribute costs through scaled production; Promoting Quality by design (QbD) principles and automated closed systems to reduce operational errors and contamination risks, aiming for systematic breakthroughs in GMP‐related bottlenecks and accelerating the clinical translation of hDPSCs [185].
④ Limited synaptic integration. Although multi‐step induction (epigenetic priming → EGF/β‐FGF/retinoic acid neuralization → BDNF/GDNF maturation) enhances neural marker expression, many hDPSCs‐derived neurons remain electrophysiologically immature. They frequently lack stable voltage‐gated Na^+^/K^+^ currents, depend mainly on Ca^2+^ conductance, and exhibit limited repetitive APs. Insufficient Synapsin expression and sparse chemical synapse formation restrict durable circuit integration, which may explain the predominantly short‐term functional benefits reported in vivo [68].
⑤ Immunogenicity and tumorigenic risk. The safety concerns surrounding the clinical translation of hDPSCs encompass two major aspects: immunogenicity and tumorigenic risk. Long‐term safety remains to be further validated through follow‐up studies. Regarding immunogenicity, as MSCs, hDPSCs generally exhibit low immunogenicity. They display low MHC‐I expression and negligible MHC‐II and co‐stimulatory molecule expression, making them difficult for T cells to recognize and trigger strong immune rejection. They also exert significant immunosuppressive effects by inhibiting T and B cell proliferation, regulating dendritic cell and NK cell function, and releasing immunomodulatory factors. In animal studies, allogeneic hDPSC transplantation showed no significant rejection reactions. Allogeneic transplantation in dogs successfully achieved pulp regeneration, and allogeneic therapy in patients with periodontal defects also showed no rejection. Early clinical trials also demonstrated that hDPSCs administration was generally well tolerated, with only mild, reversible adverse reactions observed [47]. Regarding tumor risk, non‐genetically modified hDPSCs exhibit extremely low tumorigenicity. Clinical‐grade mobilized dental pulp stem cells (MDPSCs) do not form tumors in immunodeficient mice and maintain stable karyotypes [186]. Although immortalized hDPSCs constructed using methods such as human telomerase reverse transcriptase (hTERT) can be expanded long‐term, the literature emphasizes that their long‐term genetic stability and potential tumorigenic risks remain unclear, necessitating ongoing monitoring. Consequently, their current clinical application is limited [187]. To mitigate these risks, clinical translation should prioritize the use of primary or limited‐passage cells, implement GMP‐grade standardized preparation, exercise caution with genetic modification strategies, and consider cell‐free therapies such as EVs. Concurrently, enhanced long‐term follow‐up is essential to maximize efficacy while minimizing immunological and tumorigenic risks.

## 7. Future Directions

Preclinical evidence indicates that despite the low survival rate of transplanted hDPSCs and limited engraftment in neurogenic niches, significant symptomatic improvements in neurodegenerative models are consistently observed. These findings shift the therapeutic paradigm from cell replacement to a minimal effective cell dose coupled with potent paracrine activity. To harness this potential, future research must prioritize the standardization of hDPSCs isolation and secretome (including EVs) preparation protocols to ensure the reproducibility and potency of these biological factories. The therapeutic efficacy of hDPSCs is highly sensitive to in vitro expansion conditions. Prolonged cultivation often leads to a decline in stemness, increased expression of senescence markers, and lineage bias, which collectively diminish the regenerative “cargo” within their secretome. Consequently, strict quality control standards must be established to limit cumulative population doublings and define upper thresholds for senescence indices. Furthermore, the transition to serum‐free, xeno‐free, and cGMP‐compliant culture systems is essential to maintain neural potency while eliminating the immunogenicity and batch‐to‐batch variability associated with FBS. To bridge the gap between bench and bedside, future release standards for hDPSCs products should move beyond basic immunophenotyping toward functional assays directly linked to neural recovery. Quantitative assessment of neuronal differentiation efficiency, neurotrophic factor secretion profiles, and their capacity to promote neurite outgrowth or enhance neuronal survival. Validated animal model readouts that correlate cellular activity with improvements in cognitive and motor functions. While Phase I and II trials for HD have demonstrated favorable safety and preliminary motor benefits (e.g., NestaCell), these studies are constrained by small sample sizes and limited follow‐up durations [157, 158]. There is an urgent need for large‐scale, multicenter, randomized Phase III trials to confirm long‐term efficacy, and safety. Similarly, for conditions such as AD and SCI, clinical translation must iterate through multi‐site trials to optimize dosing regimens, delivery routes, and functional endpoints on the foundation of standardized manufacturing.

## 8. Conclusions

hDPSCs represent a unique and accessible source of MSCs with an inherent neural crest origin, conferring distinct advantages in neural tissue repair. Their multifaceted mechanisms—including differentiation into neural lineages, robust paracrine secretion of neurotrophic and immunomodulatory factors, and promotion of angiogenesis—contribute to functional recovery in various models of nervous system injury. Integration with advanced biomaterial scaffolds and optimized delivery techniques further enhances their therapeutic potential. Nonetheless, clinical translation of hDPSC‐based therapies is still hindered by challenges related to controlling differentiation pathways, immune compatibility, and standardization of manufacturing protocols. Continued research integrating molecular biology, bioengineering, and clinical studies is essential to address these barriers. Harnessing the full therapeutic capacity of hDPSCs will provide an innovative and promising avenue for treating currently intractable neurological disorders, advancing regenerative strategies within both neurology and dental medicine.

**Nomenclature**
ADSCs: adipose‐derived stem cells
BDNF: brain‐derived neurotrophic factor
BMSCs: bone marrow‐derived mesenchymal stem cells
CNS: central nervous system
ESCs: embryonic stem cells
FCS: fetal calf serum
hDPSCs: human dental pulp stem cells
iPSCs: induced pluripotent stem cells
ITS: insulin–transferrin–selenium
MAP2: microtubule‐associated protein 2
MHC‐II: major histocompatibility complex class II
MSCs: mesenchymal stem cells
NCAM: neural cell adhesion molecule
NGF: nerve growth factor
NSCs: neural stem cells
NF‐H: neurofilament‐H
NF‐M: neurofilament‐M
PNS: peripheral nervous system
TUBB3: tubulin beta 3 class III
VEGF: vascular endothelial growth factor
NT‐3: neurotrophin‐3
GDNF: glial cell line–derived neurotrophic factor
HGF: hepatocyte growth factor.

## Author Contributions

**Xinxuan Wang**: conceptualization, writing – original draft preparation. **Baicheng Yi**: writing – review and editing.

## Funding

This research was funded by Stable Support Project of Shenzhen (No. 20231121233246001) and Bao’an District Medical and Health Research Project (No. 2024JD104).

## Conflicts of Interest

The authors declare no conflicts of interest.

## Data Availability

No underlying data was collected or produced in this study.
